# Work from home and the association with sedentary behaviors, leisure-time and domestic physical activity in the ELSA-Brasil study

**DOI:** 10.1186/s12889-023-15167-z

**Published:** 2023-02-10

**Authors:** Patricia de Oliveira da Silva Scaranni, Rosane Härter Griep, Francisco José Gondim Pitanga, Sandhi Maria Barreto, Sheila Maria Alvim Matos, Maria de Jesus Mendes da Fonseca

**Affiliations:** 1 National School of Public Health, Oswaldo Cruz Foundation, Manguinhos, Rio de Janeiro, RJ Brazil; 2 Laboratory of Health and Environment Education, Oswaldo Cruz Foundation, Manguinhos, Rio de Janeiro, RJ Brazil; 3 Department of Physical Education, School of Education, Federal University of Bahia, Salvador, Brazil; 4 School of Medicine & Clinical Hospital, Federal University of Minas Gerais, Belo Horizonte, MG Brazil; 5 Institute of Collective Health, Federal University of Bahia, Salvador, BA Brazil

**Keywords:** Work from home, Sedentary behaviors, Physical activity, Household work

## Abstract

**Background:**

Work from home (WFH) can impact workers´ sedentary behaviors and levels of physical activity. The aim of this study was to estimate the association between WFH and workers´ sedentary behaviors, leisure-time and domestic physical activities during the COVID-19 pandemic and verify whether age and sex may act as effect modifiers.

**Methods:**

We conducted a cross-sectional study of 2544 participants in the supplementary study on COVID-19 in the Longitudinal Study of Adult Health (ELSA-Brasil) from July 2020 to February 2021. We assessed screen time (≤ 8 h/day *versus* > 8 h/day), accumulated sitting time (≤ 8 h/day versus > 8 h/day) as sedentary behaviors on a typical day, and leisure-time (active versus inactive, according to World Health Organization recommendations) and domestic (low versus high, according to median) physical activity, using the *International Physical Activity Questionnaire* (IPAQ), before and during social distancing. Logistic regression models were used.

**Results:**

Participants that were working from home during social distancing showed increased odds of screen time and sitting time greater than 8 h/day (OR = 3.12; 95%CI: 2.32–4.20 and OR = 2.68; 95%CI: 2.02–3.56, respectively) and higher odds of high domestic physical activity (OR = 1.29; 95%CI: 0.99–1.67) when compared to those not working from home. There was no association between WFH and leisure-time physical activity (OR = 0.99, 95%CI: 0.75,1.31). Age was an effect modifier in the association between WFH and leisure-time physical activity and domestic activity. Older people working from home showed higher odds of physical inactivity (OR = 1.84, 95%CI: 1.07,3.16) and high domestic physical activity (OR = 1.92, 95%CI: 1.12,3.27) compared to older people not working from home.

**Conclusion:**

WFH was associated with sedentary behavior > 8 h/day and high domestic physical activity. In the older people, WFH was associated with physical inactivity and high domestic physical activity. As sedentary behavior and physical inactivity are consistently negatively associated with health, it is important to discuss policies to manage WFH that allow pauses from physical activities and performance of hours of work within preestablished limits to reduce sedentary behavior. In addition, individuals working from home, especially the older people, should be encouraged to engage in leisure-time physical activity as a form of health promotion.

## Background

Work from home (WFH) has gained considerable importance due to progress in information technology and globalization [1]. WHF has intensified and been reaffirmed as a practical tool for workers´ protection and for maintenance of economic activity in the context of the crisis caused by the COVID-19 pandemic [2].

The literature has reported mixed results on the advantages and disadvantages of remote work. Some studies point to advantages such as a reduction in commuting time, greater autonomy and flexibility, greater productivity, and better balance between work and personal life [1, 3]. Other studies associate WFH with greater volume and intensity of work and thus a longer and heavier workday compared to conventional work [4, 5], potentially limiting the time for activities that preserve or improve health while promoting sedentary behaviors and physical inactivity [1, 3, 4].

Sedentary behavior is defined as any waking behavior in which energy expenditure is less than 1.5 metabolic equivalents of task (METs), while sitting, reclining, or lying down [6] and that can be measured by screen time and accumulated sitting time [7]. Such behavior has been associated with all-cause mortality, cardiovascular diseases, and increased body mass index, waist circumference, plasma lipids, and blood pressure [8–10].

Physical inactivity is defined as failure to comply with recommendations for physical activity [6]. It is associated with the development of various chronic noncommunicable diseases, all-cause and cardiovascular mortality [6], and more recently, increased risk of severe outcomes from COVID-19 (hospitalization, ICU admission, and death) [11]. Despite acknowledgement of the health benefits of physical activity, the prevalence of physical inactivity is growing. According to estimates from 2016, 27.5% of the world population fail to reach recommended levels of physical activity [12], a situation that was intensified during social distancing with the closing of gyms, clubs, and parks [13, 14]. The decline in physical activity over the years has also reached the workplace, with an increase in sedentary occupations and the possibility of working from home [15]. Considering that more sedentary individuals at work tend to be more sedentary outside of work hours, interventions in work are important for increasing energy expenditure [16].

Meanwhile, domestic activity accounts for 35.6% of moderate to vigorous physical activity (MVPA), especially in women and the older people, and can contribute to sufficient levels of physical activity to stay healthy [17, 18]. Domestic activity can include housekeeping, gardening, and other household chores and has been associated with a reduction in all-cause mortality in less active individuals [19]. Particularly during the pandemic, the recommendation to stay at home and the need for social distancing may have limited the presence of domestic workers in the households, potentially influencing the time devoted to domestic physical activities related to housekeeping and cleaning.

Although social distancing during the COVID-19 pandemic has been associated with longer screen time and sitting time and less leisure-time physical activity [13, 14, 20], it is not completely clear whether WFH could also be responsible for these behaviors. Meanwhile, the association between WFH and domestic physical activity has still not been assessed.

The current study aimed to estimate the association between WFH and sedentary behaviors, leisure-time and domestic physical activity in workers in the Longitudinal Study of Adult Health and verify whether age and sex may act as effect modifiers.

## Methods

### Study population and design

**Fig. 1:**
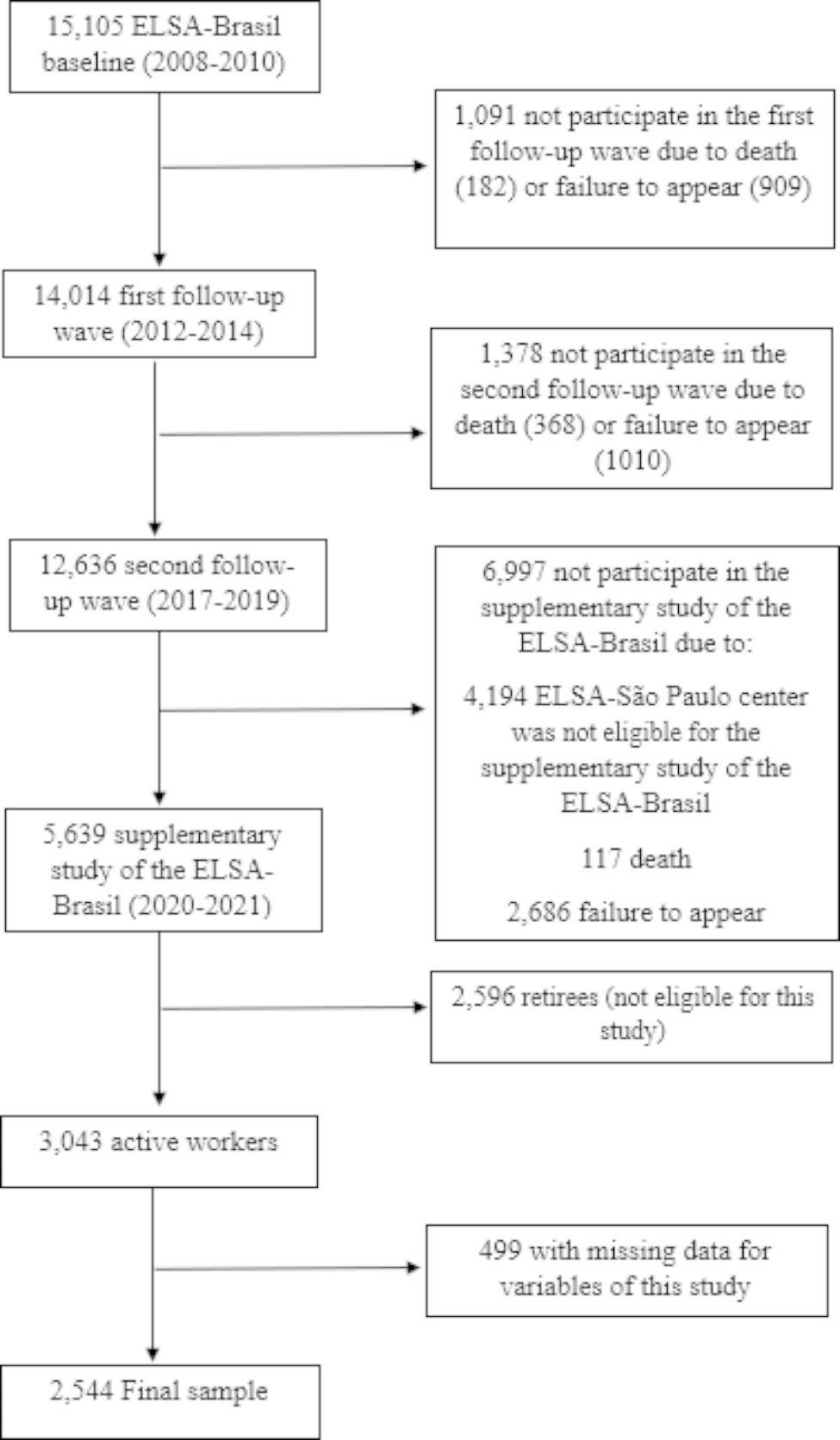
Flowchart of participants in the present study

This cross-sectional study used data from the supplementary study of the Longitudinal Study of Adult Health (ELSA-Brasil) to assess the short- and long-term impacts of COVID-19. ELSA-Brasil is a multicenter cohort consisting of actively working or retired civil servants from teaching and research institutions in six Brazilian state capitals (São Paulo, Belo Horizonte, Porto Alegre, Rio de Janeiro, Salvador, and Vitória). The cohort study seeks to contribute relevant information on the development of chronic noncommunicable diseases, particularly cardiovascular diseases and diabetes [21]. Details on the sampling, recruitment, and selection in ELSA-Brasil have been published elsewhere [22]. The baseline occurred in 2008–2010 and included 15,105 participants with 35 to 74 years of age who underwent clinical examinations and interviews. Participants returned to the corresponding study sites for the first and second follow-up waves in 2012–2014 (n = 14,014) and 2017–2019 (n = 12,636), respectively. From July 2020 to February 2021, participants in the second follow-up wave (n = 12,636), except for those from São Paulo (n = 4194), were invited to participate in the supplementary study, such that 5639 participants (66,79%) answered questionnaires by cellphone or computer, using an app developed especially for the study. We used data from 3043 (54%) active workers, and of these we excluded those who lacked complete information on WFH, sedentary behaviors, physical activity, and domestic activity (499). This study´s sample thus consisted of 2544 participants (1247 men and 1297 women), as shown in Fig. 1.

### Exposure: work from home

WFH was assessed with the following question: “Since the beginning of social distancing, have you done work from home?” The options were “no” or “yes”.

Outcomes: Sedentary behavior, leisure-time and domestic physical activity.

Participants were asked questions on the number of hours they spent in front of any screen (including cell phone, computer, TV, laptop, or others) and the number of hours they spent sitting down, reclining or lying down daily, excluding hours of sleep (cumulative sitting time) after social distancing on a typical day. We used the cutoff point of 8 h/day for each of these sedentary behaviors individually. Then, subjects were classified as having ≤ 8 h/day of screen time (reference) versus > 8 h/day and ≤ 8 h/day of cumulative sitting time (reference) versus > 8 h/day, according to Meyer et al.[23], because 8 h is the most common workday length in Brazil´s public service. This same cutoff of 8 h/day also coincides with the median of cumulative sitting time and screen time in our study population after social distancing. Sedentary behaviors had already been explored in the first follow-up wave of ELSA-Brasil [7].

Physical activity was measured in two domains: leisure-time and domestic domains with the *International Physical Activity Questionnaire* (IPAQ), long version, consisting of questions on the frequency and duration of these physical activities performed in minutes/week [24]. The leisure-time domain includes activities that participants perform in their free time: walking (not related to work), medium/moderate activities (swimming or pedaling at a moderate pace, practicing sports for fun, etc.), and vigorous physical activities (running, gym workouts, fast pedaling, competitive sports, etc.). Based on the leisure-time domain of physical activity, participants were classified as physically active (reference) and inactive (< 150 min/week of moderate physical activity or < 75 min/week of vigorous physical activity or < 150 min/week of a combination of the equivalent of both intensities - MVPA), according to World Health Organization recommendations [25].

Meanwhile, the domestic domain includes moderate activities at home (carrying light weights, washing windows, sweeping, mopping the floor), medium/moderate activities in the garden or yard (carrying light weights, washing windows, sweeping, raking), and finally vigorous physical activities in the garden or yard (weeding the garden, cleaning the yard, etc.). Participants were classified according to the median in minutes/week spent in these activities (207.5 min/week) as low (reference) versus high domestic physical activity.

### Covariables

The covariables were age (in years and age group: <60 years - adults; ≥60 years – older people), sex (male; female), schooling (Master´s/PhD; undergraduate/specialization; secondary school or less), race/ethnicity (white; mixed; black), marital status (without spouse/partner; with spouse/partner), per capita income (US$), and smoking (non-smoker/former smoker; smoker), all selected a priori according to the literature. Participants were also asked about screen time, accumulated sitting time, and time spent in leisure-time and domestic physical activity before social distancing, and these variables were treated the way as in the outcome. These last variables were possible to obtain because there was an option in each question of the IPAQ to answer about the time spent in sedentary behaviors and leisure-time and domestic physical activity before and during the pandemic.

### Data analysis

We conducted descriptive analyses through absolute and relative frequencies for qualitative variables and means and standard deviations for quantitative variables. We estimated logistic regression models, expressed as odds ratios (OR) and 95% confidence intervals (95%CI) to assess the association between WFH and sedentary behaviors, leisure-time and domestic physical activity during the pandemic. The covariables were chosen a priori based on the literature [26–28] and on bivariate analyses from which variables with p ≤ 0.20 were included in the multivariate logistic regression. A forward approach was used to choose the final model, and variables with p ≤ 0.05, were retained.

We used the Akaike information criterion (AIC) to assess the models´ quality, with lower values indicating better fit. Collinearity between the covariables, specifically between education and income, was tested with the generalized variance inflation factor (VIF), with values greater than 10 indicatives of collinearity, but it was not observed (VIF < 10).

We performed unadjusted (models 1) and adjusted models. The models pertaining to screen time and accumulated sitting time were adjusted for age and education (models 2) and their respective sedentary behaviors before social distancing (models 3). Meanwhile, the models pertaining to leisure-time and domestic physical activity were adjusted for age, sex, education and income (models 2) and for leisure-time and domestic physical activity before social distancing (models 3).

We opted to include sedentary behaviors and leisure-time and domestic physical activity prior to the pandemic since it has been demonstrated that pre-pandemic habits may have impacted changes induced by social distancing [29]. We tested multiplicative interaction between WFH and age group (< and ≥ 60 years) and sex, including an interaction term in the final model, because, according to the literature, gender and age are variables that could modify the effect of WFH on sedentary behaviors and, mainly, on leisure-time and domestic physical activity. Therefore, we performed stratified analyses for the significant interactions (p < 0.05). The R software version 4.0.4 was used for all the analyses.

## Results

Of the 2544 study participants, 80% performed WFH. Most had higher education (Master´s/PhD), reported white race/ethnicity, were nonsmokers or former smokers, and had higher income compared to those not performing WFH. There was no statistically significant difference between participants that performed WFH and those performing regular work in terms of sex, marital status, or age (Table 1).

**Table 1:** Characteristics of the study population according to WFH. Supplementary study on COVID-19, ELSA-Brasil (2020–2021), n = 2544

	Work from home (WFH)			
	No	Yes	Total	P-value*
**Total**	556	1988	2544	
**Age (years) - mean (SD)**	55.4 (7.0)	55.2 (7.4)	55.2 (7.3)	0.428
**Age group – n (%)**				
Adults (< 60 years)	390 (21.1)	1454 (78.9)	1844	0.162
Older people (≥ 60 years)	166 (23.7)	534 (76.3)	700	
**Sex – n (%)**				0.268
Male	261 (20.9)	986 (79.1)	1247	
Female	295 (22.7)	1002 (77.3)	1297	
**Schooling – n (%)**				< 0.001
Master´s/PhD	60 (5.2)	1085 (94.8)	1145	
Undergraduate/specialization	182 (21.3)	672 (78.7)	854	
Secondary or less	308 (58.1)	222 (41.9)	530	
**Income** (US$) **- mean (SD)**	608.2 (510.3)	1020.5 (675.9)	930.4 (665.5)	< 0.001
**Race/ethnicity – n (%)**				< 0.001
White	225 (15.6)	1220 (84.4)	1445	
Mixed	207 (27.9)	535 (72.1)	742	
Black	106 (36.6)	184 (63.4)	290	
**Marital status – n (%)**				0.795
With spouse/partner	339 (21.1)	1270 (78.9)	1609	
Without spouse/partner	178 (21.5)	649 (78.5)	827	
**Smoking – n (%)**				0.01
Nonsmokers/former smokers	506 (21.3)	1870 (78.7)	2376	
Smokers	50 (29.8)	118 (70.2)	168	
**Screen time, pre-COVID-19 – n (%)**				< 0.001
≤ 8 h/day	521 (25.3)	1542 (74.7)	2063	
> 8 h/day	35 (7.3)	446 (92.7)	481	
**Screen time during COVID-19 – n (%)**				< 0.001
≤ 8 h/day	479 (31.3)	1052 (68.7)	1531	
> 8 h/day	77 (7.6)	936 (92.4)	1013	
**Sitting time, pre-COVID-19 – n (%)**				
≤ 8 h/day	479 (25.1)	1430 (74.9)	1909	< 0.001
> 8 h/day	77 (12.1)	558 (87.9)	635	
**Sitting time during COVID-19 – n (%)**				< 0.001
≤ 8 h/day	438 (31.5)	952 (68.5)	1390	
> 8 h/day	118 (10.2)	1036 (89.8)	1154	
Leisure-time physical activity, **pre-COVID-19 – n (%)**				< 0.001
Active	156 (16.4)	797 (83.6)	953	
Inactive	400 (25.1)	1191 (74.9)	1591	
Leisure-time physical activity **during COVID-19 – n (%)**				< 0.001
Active	111 (16.8)	551 (83.2)	662	
Inactive	445 (23.6)	1437 (76.4)	1882	
**Domestic physical activity, pre-COVID-19 – n (%)**				< 0.001
Low	192 (15.7)	1034 (84.3)	1226	
High	364 (27.6)	954 (72.4)	1318	
**Domestic physical activity during COVID-19 – n (%)**				< 0.001
Low	242 (19.0)	1030 (81.0)	1272	
High	314 (24.7)	958 (75.3)	1272	

*Student’s t-test for continuous variables and Pearson’s chi square test for categorical variables

Participants reporting WFH displayed significantly higher rates of screen time and accumulated sitting time greater than eight hours/day and lower rates of physical inactivity and high domestic physical activity (pre- and post-COVID-19) when compared to participants not doing WFH (Table 1).

After adjusting for covariables, participants performing WFH during social distancing showed three-fold significantly higher odds of screen time and accumulated sitting time greater than eight hours a day and 29% higher odds (with borderline significance) of high domestic physical activity (OR = 1.29, 95%CI: 0.99–1.67) compared to those not performing WFH. There was no association between WFH and leisure-time physical activity (Table 2).

**Table 2:** Association between WFH, sedentary behaviors, physical inactivity, and domestic physical activity during the COVID-19 pandemic

				Screen time > 8 h/day (OR and 95%CI)		Sitting time > 8 h/day (OR and 95%CI)		Physical inactivity (OR and 95%CI)		High domestic physical activity (OR and 95%CI)		
Model 1		Does not perform WFH		1		1		1		1		
		Performs WFH		**5.53 (4.28,7.15)**		**4.04 (3.24,5.04)**		**0.65 (0.52,0.82)**		**0.72 (0.59,0.87)**		
		AIC		3200.4		3331.2		2906.7		3518.8		
Model 2		Does not perform WFH		1		1		1		1		
		Performs WFH		**4.19 (3.17,5.54)** ^**a**^		**2.98 (2.33,3.81)** ^**a**^		0.93 (0.72,1.22)^b^		1.14 (0.91,1.43)^b^		
		AIC		3109.2		3257.9		2839.6		3333.3		
Model 3		Does not perform WFH		1		1		1		1		
		Performs WFH		**3.12 (2.32,4.2)** ^**c**^		**2.68 (2.02,3.56)** ^**d**^		0.99 (0.75,1.31)^e^		**1.29 (0.99,1.67)** ^**f**^		
		AIC		2716.1		2644.8		2579.5		2715.0		

WFH: work from home; OR: odds ratios; CI: confidence interval; AIC: Akaike information criterion

Model 1: unadjusted model

^a^Model 2: model 1 + age + schooling

^b^Model 2: model 1 + age + sex + schooling + income

^c^Model 3: model 1 + age + schooling + pre-COVID screen time

^d^Model 3: model 1 + age + schooling + pre-COVID sitting time

^e^Model 3: model 1 + age + sex + schooling + income + pre-COVID leisure-time physical activity

^f^Model 3: model 1 + age + sex + schooling + income + pre-COVID domestic physical activity

Values in bold indicate statistical significance

Age was an effect modifier of WFH on leisure-time (p = 0.00984) and domestic (p = 0.035412) physical activity. (Table 3). Older people working from home showed 84% higher odds (OR = 1.84, 95%CI: 1.07–3.16) and 92% higher odds (OR = 1.92, 95%CI: 1.12–3.27) of physical inactivity and high domestic physical activity, respectively, compared to older people performing regular professional work (Table 4).

**Table 3:** Interactions between WFH and sex and age in sedentary behaviors, leisure-time and domestic physical activity

Interactions	Screen time		Cumulative sitting time	Leisure-time physical activity	Domestic physical activity
WFH*age	0.11468		0.07375	0.00984**	0.035412*
WFH*sex	-		-	0.53986	0.2259

WFH: work from home; Values in bold indicate statistical significance; * p < 0.05; ** p < 0.01

**Table 4:** Association between WFH and physical inactivity and domestic physical activity in adults and older people

		Physical inactivity (OR and 95%CI)		High domestic physical activity (OR and 95%CI)	
		Adults (n = 1844)	Older people (n = 700)	Adults (n = 1844)	Older people (n = 700)
Model 1	Does not perform WFH	1	1	1	1
	Performs WFH	**0.55 (0.41,0.72)**	0.97 (0.65,1.45)	**0.61 (0.49,0.77)**	1.02 (0.72,1.44)
	AIC	2111.9	793.4	2541.1	971.4
Model 2	Does not perform WFH	1	1	1	1
	Performs WFH	0.77 (0.56,1.05)^a^	1.55 (0.93,2.58)^a^	0.97 (0.75,1.27)^c^	**1.60 (1.01,2.54)** ^**c**^
	AIC	2066.0	777.2	2413.7	899.5
Model 3	Does not perform WFH	1	1	1	1
	Performs WFH	0.79 (0.56,1.10)^b^	**1.84 (1.07,3.16)** ^**b**^	1.10 (0.81,1.49)^d^	**1.92 (1.12,3.27)** ^**d**^
	AIC	1887.6	697.2	1970.0	739.8

WFH: work from home; OR: odds ratios; CI: confidence interval; AIC: Akaike information criterion

Model 1: unadjusted model

^a^ Model 2: model 1 + age + sex + schooling + income

^b^ Model 2: model 1 + age + sex + schooling + income + pre-COVID leisure-time physical activity

^c^Model 3: model 1 + age + sex + schooling + income

^d^Model 2: model 1 + age + sex + schooling + income + pre-COVID domestic physical activity

Values in bold indicate statistical significance

## Discussion

In this study, WFH was associated with higher odds of screen time and accumulated sitting time equal to or greater than eight hours/day and higher odds of high domestic physical activity. These associations were independent of these behaviors before the pandemic. No association was observed between WFH and leisure-time physical activity. Age was an effect modifier in the analyses of leisure-time and domestic physical activity. In the analyses stratified by age, older people individuals working from home showed increased odds of physical inactivity and high domestic physical activity.

The results for screen time and accumulated sitting time are consistent with the literature. McDowell et al. [26] found longer screen time (β = 34.15 min/day, p < 0.001) and accumulated sitting time (β = 30.93 min/day, p < 0.001) associated with WFH during the COVID-19 pandemic in 2,303 employees of Iowa State University. The same was observed in exploratory studies that compared mean screen time between individuals with and without WFH during social distancing [27, 28].

The fact that individuals that WFH show higher odds of sedentary behaviors exceeding eight hours/day may indicate that the workday at home is actually longer, as indicated in other studies [4, 5], considering that eight hours is the normal cutoff for the expected workday in public service.

Social distancing and lockdown measures per se increase individuals´ dependence on various electronic devices that allow communication, resulting in longer screen time [27]. WFH uses information and communication technologies (ICTs) extensively via screens that induce more static activities, generally sitting, resulting to lower energy expenditure [1, 4, 30]. Persons who began working from home would thus be expected to display more sedentary behaviors. Research has also shown that in this new work context, the workday is often longer, online meetings are more frequent, and there are fewer opportunities to engage in activities away from home or the office, a situation intensified by restrictions due to COVID-19 and that exacerbated occupational sedentarism [26].

A study on the time trend in total physical activity in Brazil reported that the occupational domain accounted for the largest absolute decline [15]. Studies report that sedentary occupations have increased by 83% since 1950 [31] and that sedentary time represents 81.8% of the work hours in conventional fulltime office jobs [30]. Recent Brazilian estimates based on data from the Telephone Survey on Risk and Protective Factors for Chronic Diseases (VIGITEL survey) corroborated this information by estimating only 45.2% prevalence of physical activity at work in the year 2016 [32]. According to a study in 14 countries, including Brazil, during the pandemic the decline in physical activity in the occupational domain was greater than in leisure time, namely 10% for vigorous physical activity and 20% for MVPA [14].

As for leisure-time physical activity, the fact that we did not find a statistically significant association may be explained by the restrictions on circulation resulting from social distancing that impacted opportunities for physical activity by all individuals equally, independently of work modalities (in-person versus remote) [14, 26]. In addition, the study population in ELSA-Brasil was already quite physically inactive before the pandemic [7]. These factors make the study population more homogeneous in relation to leisure-time physical activity, hindering the appearance of associations. The time saved by individuals working from home might have facilitated physical exercise, but given the pandemic´s restrictions, physical activity away from home was often hampered or prevented entirely, especially during the more critical lockdown periods.

McDowell et al. [26] also found no association between WFH and leisure-time physical activity in study with US adults during the COVID-19 pandemic. In a Brazilian study conducted by Brusaca et al. [33], office workers working from home during social distancing were found to have reduced their physical activity practice in leisure time when comparing the periods before the pandemic, with 11% decrease for light physical activity (LPA) and 42% decrease for MVPA). On the other hand, a recent Brazilian study with data from 1,750 volunteers showed that WFH was a protective factor for physical inactivity during the COVID-19 pandemic [34].

According to the age-stratified analyses, older people working from home showed higher odds of physical inactivity compared to those working in person. There are no studies on the association between WFH and physical activity that compare younger and older adults. The literature has documented extensively that older adults tend to be more physically inactive than the younger population [35]. However, studies on physical activity during COVID-19 showed that younger individuals were more prone to reduce their levels of leisure-time physical activity when compared to the older people [20, 36], and one study found a U-shaped relationship in this association, that is, the youngest and oldest adults showed the highest reduction in physical activity [14].

Although the recommendations for physical activity in younger adults also apply to the older people, it is recommended that older individuals should perform multicomponent physical activity, that is, with aerobic exercise, muscle strengthening, and balance training in a single session, with moderate intensity or greater, three days a week or more, to increase functional capacity, improve bone health, and prevent falls [6].

Considering the low energy expenditure induced by WFH, the balance between periods of sedentary behavior and activities that generate energy expenditure is essential, since these habits are mutually exclusive, and at the same time there is a relationship of co-dependence, i.e., time spent in sedentary behavior necessarily influences the time left for activities with energy expenditure and vice versa [37, 38]. In practical terms, this means that the more time an individual spends in sedentary behaviors, the fewer the opportunities for physical activity. The adverse consequences of sedentary behaviors may outweigh the benefits of physical activity [38]. To organize a more active and healthier day, it is thus necessary to consider sedentary behaviors as components of the 24 h [6]. Therefore, the World Health Organization recently recommended [6] replacing sedentary time with some physical activity of any intensity, including light intensity, since health benefits have been demonstrated from physical activity even below the recommended levels (besides involving targets that are easier to achieve) [19, 37, 38].

Especially during the pandemic when physical activity has been inhibited to a large extent, it has been necessary to reduce sedentary behavior, replacing it with activities that are feasible for persons working from home. There are leisure-time physical activities that can be performed in the home environment, even without special equipment and with limited space, such as walking short distances, dancing, climbing stairs, playing with children, standing or walking around the house while talking on the phone, skipping rope, and doing squats, sit-ups, and strength and aerobic exercises [14, 29, 39].

As far as we know, this was the first study to assess the domestic domain of physical activity associated with WFH, and our results showed a protective effect. We consider it important to highlight this association, even if borderline, due to its relatively high magnitude (OR = 1.29; 95%CI: 0.99–1.67). Furthermore, a borderline significance does not necessarily mean that there is no association because it is more likely that the true effect to be around the estimated measure of association (in our case, OR = 1.29) than at either extremes of the confidence interval, according to Hackshaw & Kirkwood [40]. The borderline statistical significance may be due to a more diluted association by age because our age-stratified analyses showed this association only in the older people working from home who are few in our population compared to adults, and somehow compensated for their physical inactivity by performing domestic activities.

Domestic activities during the pandemic may be a way of contributing to levels of physical activity, considering the decline in leisure-time and occupational physical activity and the increase in sedentary behaviors. A study of the adult population in Singapore highlighted that 44 min/day of mixed domestic activities are equivalent to 150 min of walking/week and that even light-intensity domestic activities, jointly or when performed for longer periods, can contribute significantly to the total amount of physical activity [41].

However, in the context of the pandemic, given the need for social distancing and staying at home, many people may have dismissed their domestic workers and performed their own household chores [18]. Still, we should point out that since this study was conducted over the course of the COVID-19 pandemic, there were participants who answered the questionnaire at different moments, ranging from the more critical moments (when it was not advisable to maintain domestic workers at home) to the more flexible times in which it was already possible to rely on such services. This may have impacted our estimates since it was not possible to observe a large increase in the proportions of domestic physical activity during the pandemic compared to the pre-COVID-19 period among those performing WFH in our descriptive analyses (Table 1). Likewise, we lack information on whether domestic workers remained in service during the pandemic, which might better explain the observed association between WFH and domestic physical activity.

The literature shows that domestic work is unequal between the sexes, making WFH an additional challenge for women, especially those with small children, facilitating their exclusive return to their roles as traditional homemakers [42, 43]. However, in our study the sex variable was not an effect modifier (Table 3). Women may have maintained their same domestic activities as before the pandemic or increased them very little, and men may have begun to perform such activities during the pandemic, thus tending to smooth out any major disparities.

This study´s strengths feature the use of a large-scale population sample and the exploration of the domestic domain of physical activity. We have added evidence that was previously scarce and used cutoff points based on the literature and on the prevailing recommendations on leisure-time physical activity. Another advantage is that given the nature and specificity of the exposure and outcome, it was possible to clearly identify the direction of the temporality, thus allowing to rule out the possibility of reverse causality, which cannot be affirmed in most cross-sectional studies.

However, the study´s limitations include the fact that sedentary behaviors were self-reported, particularly on physical activity, so there may be an information bias. Still, we used an internationally validated instrument, the IPAQ. We also did not distinguish between screen times and accumulated sitting time performed on weekdays versus weekends, or whether they were related to leisure time or specifically to work. Likewise, we do not rule out a selection bias, since the sample was obtained by voluntary completion of the questionnaire via an app, which may explain the predominance of participants with more schooling. Still, this limitation can also be seen in other studies on the topic that used convenience sampling and similar strategies during social distancing [18, 26–28].

Our results highlight the importance of discussing policies for managing WFH that allow pauses for physical activities and completion of work hours within the preestablished limits to reduce sedentary behavior. Despite performing domestic physical activity, individuals working from home, especially the older people, should be encouraged to perform leisure-time physical activity as a form of health promotion.

## Data Availability

The datasets used and/or analysed during the current study are available from the corresponding author on reasonable request.

## Abbreviations

WFH: Work from home
ELSA-Brasil: Longitudinal Study of Adult Health
METs: metabolic equivalents of task
MVPA: moderate to vigorous physical activity
IPAQ: International Physical Activity Questionnaire
OR: odds ratios
AIC: Akaike information criterion
VIGITEL: Telephone Survey on Risk and Protective Factors for Chronic Diseases
